# Supplementary material for: Th2 Suppression Through Antigen Liver Expression Using mRNA-LNP Technology
Source: Biomedicines. 2025 Sep 19;13(9):2297. doi: 10.3390/biomedicines13092297 (PMC12467278; doi:10.3390/biomedicines13092297)
Supplement: Supplementary file 1 [file biomedicines-13-02297-s001.zip › biomedicines-3813392-supplementary.pdf]

Supplementary information

OVA-B7-1 mRNA sequence.
augggcuccaucggcgcagcaagcauggaauuuuguuuugauguauucaaggagcucaaaguccaccaugccaaugagaacaucu
ucuacugccccauugccaucaugucagcucuagccaugguauaccugggugcaaaagacagcaccaggacacagauaaauaaggu
uguucgcuuugauaaacuuccaggauucggagacaguauugaagcucaguguggcacaucuguaaacguucacucuucacuuag
agacauccucaaccaaaucaccaaaccaaaugauguuuauucguucagccuugccaguagacuuuaugcugaagagagauacccaa
uccugccagaauacuugcagugugugaaggaacuguauagaggaggcuuggaaccuaucaacuuucaaacagcugcagaucaagc
cagagagcucaucaauuccuggguagaaagucagacaaauggaauuaucagaaauguccuucagccaagcuccguggauucucaa
acugcaaugguucugguuaaugccauugucuucaaaggacugugggagaaaacauuuaaggaugaagacacacaagcaaugccuu
ucagagugacugagcaagaaagcaaaccugugcagaugauguaccagauugguuuauuuagaguggcaucaauggcuucugaga
aaaugaagauccuggagcuuccauuugccagugggacaaugagcauguuggugcuguugccugaugaagucucaggccuugagc
agcuugagaguauaaucaacuuugaaaaacugacugaauggaccaguucuaauguuauggaagagaggaagaucaaagugu acuu
accucgcaugaagauggaggaaaaauacaaccucacaucugucuuaauggcuaugggcauuacugacguguuuagcucuucagcc
aaucugucuggcaucuccucagcagagagccugaagauCucucaagcuguccaugcagcacaugcagaaaucaaugaagcaggca
gagaggugguagggucagcagaggcuggaguggaugcugcaagcgucucugaagaauuuagggcugaccauccauuccucuucu
guaucaagcacaucgcaaccaacgccguucucuucuuuggcagauguguuuccccuuaugaaguuaaacacuuggcuuuaguaaa
guuguccaucaaagcugacuucucuacccccaacauaacugagucuggaaacccaucugcagacacuaaaaggauuaccugcuuu
gcuuccgggguuucccaaagccucgcuucucuugguuggaaaauggaagagaauuaccuggcaucaauacgacaauuucccagg
auccugaaucugaauuguacaccauuaguagccaacuagauuucaauacgacucgcaaccacaccauuaagugucucauuaaauau
ggagaugcucacgugucagaggacuucaccugggaaaaacccccagaagacccuccugauagcaagaacacacuugugcucuuug
gggcaggauucggcgcaguaauaacagucgucgucaucguugucaucaucaaaugcuucuguaagcacagaagcuguuucagaa
gaaaugaggcaagcagagaaacaaacaacagccuuaccuucgggccugaagaagcauuagcugaacagaccgucuuccuuuag

Figure S1. Goblet Cell Scores.

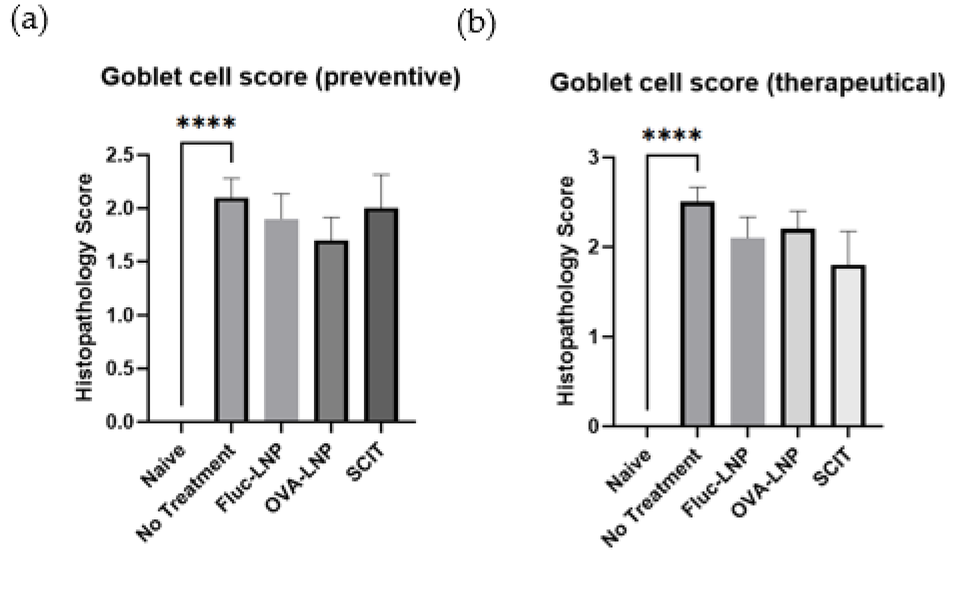

Goblet cell scores are analyzed in mouse lung (preventive administration: a, and therapeutical administration: b). Histologic lesions were graded for severity (0=absent; 1=minimal; 2=mild; 3=moderate; 4=marked; 5=severe). Group mean ± standard error of the mean (SEM). p-value was determined using t-test for comparison between no treatment group and naïve group. For the other groups to compare with no treatment group, p-value was determined using Dunn's multiple comparisons test. **** = $p<0.0001$.
